# A systematic review to understand the long-term mental health effects of influenza pandemics

**DOI:** 10.1177/14034948251391601

**Published:** 2025-11-30

**Authors:** Jessica L. Dimka, Benjamin M. Schneider, Svenn-Erik Mamelund

**Affiliations:** 1 Department of Sociology, Anthropology, and Criminal Justice, Seton Hall University, South Orange, USA; 2 Centre for Research on Pandemics & Society, Oslo Metropolitan University, Norway

**Keywords:** Pandemics, mental health, long-term health consequences, influenza

## Abstract

**Aims:**

Health effects of pandemics extend beyond morbidity and mortality from the disease itself and may include long-term mental health consequences. However, previous studies only consider narrowly defined populations at risk or examine pandemics caused by varied pathogens that may have inconsistent effects. We examine existing literature on these long-term mental health effects following one type of pandemic (influenza).

**Methods:**

We conducted a systematic review of the long-term mental health effects of the 1889, 1918, 1957, 1968, and 2009 influenza pandemics. To our knowledge, this is the first review of studies of broad populations and multiple measures of mental health morbidity.

**Results:**

The literature search returned 8190 articles. After deduplication and title/abstract and full-text screening, 12 articles were reviewed. Seven articles focused on the 1918 pandemic and five on the 2009 pandemic. Study regions were USA or North America (*n*=5), Europe (*n*=3), and Asia (*n*=4). Long-term outcomes studied were suicide (*n*=4), admission to hospital or psychiatric facility (*n*=2), stress/anxiety/post-traumatic stress disorder (*n*=4) and schizophrenia and other/related conditions (*n*=2). The suggested mechanisms were infection (*n*=6), effects of non-pharmaceutical interventions (NPIs) (*n*=3), or other exposure pathways (*n*=3). Seven studies had a moderate risk of bias and five studies a high risk of bias.

**Conclusions:**

Mental health effects have been an outcome of pandemics. Researchers should consider a variety of possible mechanisms, and that infection and restrictive NPIs may contribute to mental health morbidity. This study highlights the need for better understanding of the broader health, social, and demographic impacts of pandemics.

## Introduction

Short- and long-term impacts of pandemics extend beyond illness and death. In addition to economic, social, and political shifts, the effects of pandemics on mental health contribute to the overall outcomes. Examining long-term mental health effects of historical pandemics is necessary to grasp the potential burden and *duration* of health consequences, as well as the varied mechanisms that produce such effects. For example, illness itself might trigger or exacerbate some conditions, while socioeconomic (e.g. job or wage losses, isolation because of social distancing measures) or personal (e.g. bereavement) factors might also play a role. In this systematic review we ask the following research question: “What are the long-term effects of influenza pandemics on mental health, resulting either from illness itself or the social or economic effects of pandemics and public health responses?”.

Previous work has explored connections between epidemics or pandemics and mental health impacts, including associations of the 1918 influenza pandemic with suicide and hospitalizations [1–4], as well as psychiatric disorders following the 2003 severe acute respiratory syndrome coronavirus-1 epidemic. Research articles, commentary pieces, and the media all raised concerns about substantial short- and long-term mental health consequences of the COVID-19 pandemic [5]. Other systematic reviews have synthesized results of studies from these and other infectious disease epidemics, including Ebola, Zika, the 2009 H1N1 pandemic, and others, for example, Luo et al. [6], van der Feltz-Cornelis et al. [7], Yuan et a. [8] and Zürcher et al. [9]; see Dimka et al. [10] for a more detailed review of previous literature. Limitations of previous studies include narrow focus on specific outcomes or populations at risk; potentially confounding consideration of multiple diseases with varied causes, symptoms, and societal disruption; and for more recent pandemics, a limited ability to ascertain longer-term or post-pandemic effects. Therefore, this systematic review contributes to the literature by including studies of broad populations and multiple measures of mental health morbidity. It also takes a historical perspective that emphasizes pandemics of varying severity but with similar causative pathogens, and examines studies of long-term impacts.

In the following sections, we summarize the search, screening and review of studies using the Preferred Reporting Items for Systematic Reviews and Meta-Analyses 2020 checklist. Details of the 12 studies included in the final review are highlighted in the results section, along with an examination of trends related to location and timing of the research, and the type, duration, and direction of mental health outcomes. We conclude with a discussion of limitations in this study and areas for future research that incorporates important methodological and data considerations associated with studying historical pandemics.

## Materials and methods

The protocol for this review has been registered with the International Prospective Register of Systematic Reviews (PROSPERO) with the registration number CRD42021253307. The complete protocol also has been published [10] and is summarized here.

### Eligibility criteria

English-language studies investigating associations between influenza pandemics and long-term mental health sequelae were considered eligible if they addressed experiences of historical influenza pandemics during the study period of 1889–2009 (i.e. 1889–1890 (possibly H3N8), 1918–1920 (H1N1), 1957–1958 (H2N2), 1968–1970 (H3N2), and 2009–2010 (H1N1)). Following the PICO framework, populations (P) included both those with and those without pre-existing mental health symptoms or conditions prior to the associated pandemic. Intervention (I) is exposure of the population or participants to an influenza pandemic during the study period. Comparators or controls (C) are not relevant for this review. The review addresses outcomes (O) of mental health morbidity and related impacts, including social or economic impact, institutionalization, and/or death.

Studies were excluded if they addressed pandemic diseases besides influenza, seasonal influenza only, both seasonal and pandemic influenza without distinguishing between them, or potential mental health side effects of vaccines or treatments. Further, while eligible articles may include discussion of physical as well as mental health effects, any articles that addressed only long-term physical effects were excluded. We determined that encephalitis lethargica—especially relevant for research on the 1918 flu pandemic—was characterized primarily by physical and neurological symptoms rather than mental health symptoms and so research on this potential outcome was excluded. Further, studies linking late-in-life mental health with fetal/early exposure, and studies of mental health symptoms during influenza-like illness that resolve after acute infection (i.e. short-term effects) were also excluded. While there were no general restrictions on other study designs, case studies that address the symptoms or diagnoses of individuals, commentary or review pieces without original data, and articles on policy and/or social justice issues were ineligible.

### Information sources and search strategy

The search strategy was developed and piloted in collaboration with research librarians at Oslo Metropolitan University. Searches, which took place between January and February 2021, were conducted in Embase, MEDLINE, PsycINFO, CINAHL, Web of Science, Academic Search Ultimate, ASSIA, and Google Scholar. The final search strategy included two elements and an extra search: influenza pandemic/epidemics; mental health outcomes; and long-term effects and pandemics (in the title only). Keywords for the influenza component included specific viruses, pandemic years, and colloquial terms for different pandemics. Keywords for health-related outcomes included general terms related to mental health disorders as well as specific conditions. Time was indicated through keywords such as long term, persistent, chronic, etc. The search elements with related terms and synonyms were combined within each database, with no language or publication date restrictions applied.

### Selection process

All eligible studies (*N*=8190) were imported to EndNote and subsequently the screening program Covidence, with duplicates removed at each stage, leaving 4428 articles. The first two authors (JD and BS) independently screened titles and abstracts, and then full-text versions. At both stages of screening, discordance was assessed until a consensus was reached.

### Data collection process

The first author drafted a data abstraction form and extracted data, which was reviewed independently by the other two authors. Missing data was noted as absent for relevant individual studies.

### Data items

Extracted data, when applicable, included: 1) author and publication information; 2) sample and study details, for example, country or location, pandemic(s) considered, sample size, source of outcome data, study design including the duration or timing of follow-up; 3) outcomes (see below); and 4) independent variables or contextual details, for example, presumed cause or mechanism and demographic variables of affected individuals, if available.

### Outcomes and prioritization

The main outcomes of interest were mental health morbidity, as well as related effects of pandemics. For morbidity, potential outcomes included the mental health conditions observed, whether they were new diagnoses, when they appeared and how long they lasted, the proportion of the sample affected, and any measures of severity. Potential related impacts included, for example, whether the affected individuals were registered as disabled or received a pension, institutionalization (including whether it was voluntary and the type of institution), and/or death (including whether it resulted from suicide or other causes with mental health attributed).

### Risk of bias in individual studies

All studies were evaluated on the details of the sample or data source such as size and representativeness, and the methods or standards used for identifying mental health outcomes (e.g. diagnosed by doctor, self-reported, etc.). Additionally, quantitative studies were also evaluated based on whether studies controlled for important confounders such as age and sex; the timeframe/length of follow-up and participant attrition; and the selection and application of appropriate statistical methods. While qualitative studies were eligible, none of the studies retained for the full review used qualitative methods. The two first authors independently assessed the overall quality of each included study, with discrepancies resolved through discussion among all three authors.

### Synthesis

The results are synthesized narratively below, accompanied by tables of key characteristics of the included studies. No meta-analyses of quantitative data were performed. In the narrative review, we discuss the range of presumed causative factors and outcomes. We also discuss outcomes grouped by individual pandemic, to account for historical variation, including differences in how mental health diagnoses may be considered. We searched for disaggregation by outcomes for people with and without pre-existing mental health conditions, but this was not reported in the studies that met our eligibility criteria. We also discuss limitations and gaps to identify future research needs.

### Meta-biases and confidence in cumulative evidence

No assessment of meta-biases was performed. We determine cumulative quality below by considering the consistency of the findings.

## Results

### Study selection

**Figure 1.**
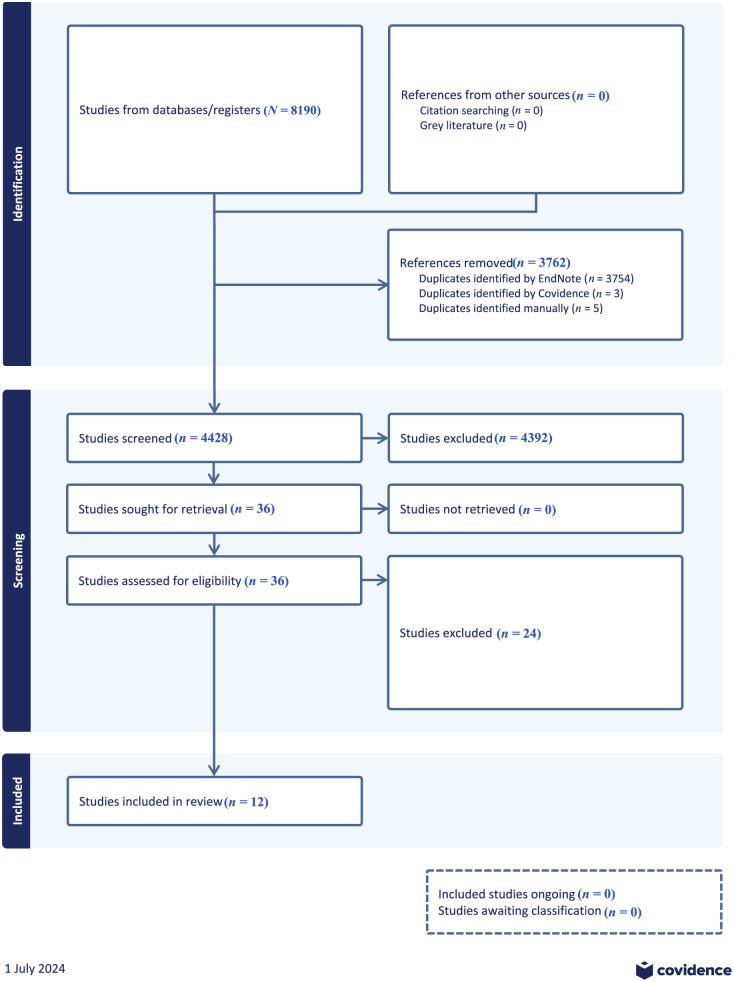
Preferred Reporting Items for Systematic Reviews and Meta-Analyses (PRISMA) flowchart for this study.

As noted above, the database search returned 8190 records, approximately half of which were identified as duplicates and removed. Of the remaining 4428 articles, 4392 were excluded during title and abstract screening based on the inclusion and exclusion criteria. After reading 36 records in the full text stage, 12 articles met criteria for the final analysis (Figure 1).

### Study characteristics

The review identified a total of 12 studies, of which seven analyzed the 1918–1920 influenza pandemic and five analyzed the 2009–2010 influenza pandemic. No included studies addressed the 1889, 1957 or 1968 pandemics. Study regions were USA or North America (five), Europe (three) and Asia (four). Long-term outcomes studied were suicide (four), admission to hospital or psychiatric facility (two), post-traumatic stress disorder (PTSD)/stress/anxiety (four) and schizophrenia and other/related conditions (two).

### Risk of bias in studies

It is likely that the strongest evidence of long-term mental health consequences of pandemics would come from studies with individual-level data for large sample sizes in which the population lived through a pandemic, and that incorporate mental health outcome measures for several months or years pre- and post-pandemic or even throughout life. None of the reviewed studies meet this ideal but perhaps unattainable study design. As indicated in Tables I and II, in which we primarily consider sample size and other details, data sources, controls for confounders, and the level of statistical analyses, most of the studies may be considered to have a moderate risk of bias. Five of the studies are considered to have a potential high risk of bias, primarily because they lack controls for confounders and/or only present descriptive statistics.

**Table I.** Reviewed studies (part 1 of 2).

Article	Pandemic	Location	Data details/level of analysis	Presumed mechanism
Wasserman 1992	1918	USA	Population—US Census Bureau and Death Registration Area. Regional coverage changed from 58.3% (1910) to 82.2% (1920) during study period.	Indirect: less social integration because of non-pharmaceutical interventions (NPIs)
Stack and Rockett 2021	1918	USA	Social distancing data from newspapers and health department reports, for 43 large cities (from Markel et al. 2007); mortality data from Census Bureau	Indirect: social distancing, independent of flu burden
Chang et al. 2020	1918	Taiwan	Population—annual Statistical Reports of Taiwan Governor-General Office, with monthly pneumonia and suicide mortality	Direct and indirect: various possibilities discussed including NPIs, effects of illness, fear of infection
Van der Heide and Coutinho 2006	1918	Netherlands	Registry for acute compulsory psychiatric admissions of Municipal Health Authority (Amsterdam); 1918 pandemic influenza mortality data	Direct: effect of illness
Delić and Plavšić 2020	1918	Austro-Hungary (now Croatia)	Registers for Pula Provincial Hospital (*n*=213 flu; *n*=200 mental illness)	Direct: effect of illness
Menninger (1926) 1994	1918	USA	Follow-up inquiries (e.g. letters) corresponding to 50 patients of Boston hospital	Direct: effect of illness
Menninger 1919	1918	USA	Boston Psychopathic Hospital records; *n*=80 (100 for some general statistics)	Direct: effect of illness
Jung et al. 2021	2009	South Korea	Data from Korea Centers for Disease Control and Prevention: influenza-like illness outpatient visitors per 1000 weekly from week 35 of 2004 to week 52 of 2017. Government surveillance system collects from 53 hospitals using geographic stratification. Mortality data from national death records of Korea National Statistical Office.	Direct: discussion considers effect of illness or subsequent development of neuropsychiatric symptoms among other potential factors
Xu et al. 2011	2009	China	Survey (*n*=1082) of university students in four provinces	Direct or indirect: general experience or exposure including media
Luyt et al. 2012	2009	France	Registry data used to identify acute respiratory distress syndrome patients to recruit for survey (*n*=37). Twelve patients received extracorporeal lung assist (ECLA).	Direct: effect of illness, especially ECLA treatment
Sprang and Silman 2013	2009	North America	Mixed methods survey distributed to parents in six US states, two Mexican cities and one Canadian city, *n*=398	Indirect: disease containment strategies (separation, quarantine, isolation)
Matsuishi et al. 2012	2009	Japan	Survey of hospital workers in Kobe, Japan, *n*=1625	Indirect: experience as health care workers, although unable to determine level of patient contact for respondents in “high-risk” environments

**Table II.** Reviewed studies (part 2 of 2).

Article	Mental health outcome	Results	Duration or timing of follow-up	Assessment of risk of bias
Wasserman 1992	Suicide	Positive and significant, suggesting pandemic increased suicide. Ordinary Least Squares (OLS) coefficient of 0.1 for effect of (influenza) mortality rate	Survey period covered 1910–1920	Moderate—inferential statistics on population-level data; models adjusted for alcohol consumption rate, lag of suicide rate, social integration during World War I, and other variables (e.g. medical doctors per population)
Stack and Rockett 2021	Suicide	Significant: a 10-unit change in social distancing index associated with a 2.9% increase in suicide rate (0.43 cases per 100,000), even when adjusted for flu mortality. Flu mortality is not a significant predictor of suicide when adjusting for social distancing.	1918 data only, so limited if any long-term implications can be determined	Moderate—inferential statistics on municipal-level data; models adjusted for flu deaths (per 100,000 population)
Chang et al. 2020	Suicide	Statistically insignificant rise in suicides during the October–December 1918 wave. A small, brief, but statistically significant increase in suicide during the second (1920) wave in January (33%, i.e. 14 excess suicides) and March (35%, i.e.,19 excess suicides).	Mortality data for 1910–1920 but more specific timing cannot be determined.	Moderate—inferential statistics on population-level data; models adjusted for month (for linear trends) and seasonality (calendar month dummies)
Van der Heide and Coutinho 2006	Admissions to psychiatric facility	Slightly higher psychiatric admissions than usual during the peak flu months of October–November 1918, but probably not significant (mean monthly rate for October 1908–1923: 26; October 1918: 32; mean monthly rate for November 1908–1932: 25.4; November 1918: 34; statistical test results not reported)	Specified as acute admissions per month, and data collection ends in February 1919	High—descriptive statistics only presented; population-level data with no adjustments or controls
Delić and Plavšić 2020	Hospitalization and diagnosis (multiple disorders including melancholia, hysteria, neurosis, psychosis, and dementia)	No apparent connection between hospitalization for flu and mental illness diagnosis	Potentially up to two years and four months (data collected 27 August 1918 to 31 December 1920; large wave of pandemic ended March 1919)	High—descriptive statistics only presented; demographic data for relatively small sample of individual records apparently collected but not used to adjust analyses
Menninger (1926) 1994	Dementia praecox/schizophrenia	Eighty percent recovered (*n*=35) or improved (*n*=5); 10% (*n*=5) unchanged; 10% (*n*=5) worse	Five-year period after initial illness/diagnosis	High—descriptive statistics only for individual-level data collected via correspondence; relatively small sample size; some changes in diagnoses before selection of follow-up sample (p.184)
Menninger 1919	Multiple; organized into four groups. Group 1: temporary delirium during acute infection, *n*=16. Group 2: dementia praecox, *n*=25. Group 3 includes a range of conditions described as “the usual psychoses”, includes manic depression, neurosyphilis, alcoholic psychoses, paranoia, etc., *n*=23. Group 4 unclassified, *n*=16.	Focus on Groups 2 and 3: mental health symptoms began on average after fever abated, ranging from less than two days to 8.5 days. Twenty-three for Group 2 (other two died) and seven for Group 3 (two died, 14 transient) categorized as “permanent” at time of follow-up.	Up to ~3 months—epidemic began in Boston 15 September and analyses consider 100 mental health patients admitted from then until 15 December 1918. Acknowledges the difficulty of determining when exactly a flu case ends.	High—descriptive statistics only on individual hospital records; minimal other demographic data or controls reported (e.g. average ages); relatively small sample size
Jung et al. 2021	Suicide	Significant positive association between influenza-like illness per 1000 and suicide, 2009–2017 (ß = 0.013, *p* = 0.018) but not before, 2004–2009 (ß = −0.066, *p* = 0.214). Relative ratios slightly but significantly higher with lags of 1–8 weeks but not after week 9.	Lag models up to 24 weeks were constructed	Moderate—inferential statistics on hospital surveillance and national statistics. Analyses adjusted for seasonality and compared with control of cancer mortality.
Xu et al. 2011	Post-traumatic stress disorder (PTSD)	Only 2% (*n* = 22) of the sample met symptomatic criteria for PTSD; two of these had been infected with flu. Regression analyses (model *R*^2^ = 0.17) showed that in North China, female gender (ß = −0.09), influenza infection of self (ß = −0.12) or contacts (ß = −0.08) and being afraid of flu (ß = −0.17) were significant predictors of stress symptoms.	Survey completed during November–December 2009, and measured frequency of stress symptoms during the past four weeks, but more specific timing cannot be determined	Moderate—inferential statistics on relatively small sample of individual survey respondents. Covariates include, for example, gender, area, university grade, knowledge about H1N1, knowing someone who was infected.
Luyt et al. 2012	Health-related quality of life, anxiety, depression, PTSD	A majority of patients had anxiety and depression symptoms and were at risk for PTSD. Fifty percent of patients in the ECLA group had severe anxiety, compared with 56% in the non-ECLA group. Twenty-eight percent of both groups had severe depression. Forty-one percent of ECLA group patients and 44% of non-ECLA group patients were at risk for PTSD.	Approximately one year (median 12.4, range 11.1–13 months)	High—inferential statistics on small sample of individual data. Covariates apparently not adjusted for in mental health outcome comparisons.
Sprang and Silman 2013	PTSD	PTSD criteria met in 30% of isolated or quarantined children, 25% of parents. Significant differences compared with parents (7% – χ^2^ = 31.411, *p* < 0.001) and children (1.1% – χ^2^ = 49.56, *p* < 0.001) who did and did not experience containment measures.	Difficult to determine: approximately one month; surveys conducted in spring 2009, with recruitment period of about one month, but at least some questions asked about frequency of symptoms during previous month	Moderate—inferential statistics on relatively small sample of individual self-selected survey respondents; adjusted for gender, age, location and disease-containment strategy
Matsuishi et al. 2012	Stress experienced during and after trauma	Exploratory factor analysis produced categories of stress, i.e. anxiety about infection, exhaustion, workload, feeling of being protected. Multiple regression results indicate the 20–29 year age group (reference *vs*. four older categories up to age 70), nurses relative to doctors (ß = 0.27), and workers in high-risk environments (ß = 0.13) had higher anxiety about infection.	Difficult to determine: first patient admitted in mid-May; return to normal practice by 8 June. Survey distributed 22 June to 31 July (~1 month after peak)	Moderate—inferential statistics and factor analysis on relatively small sample of individual survey respondents; adjusted for gender, age group, job, one of three hospital locations, risk in work environment

## Results of individual studies

Tables I and II summarize relevant details of the 12 studies, arranged in chronological order of pandemic.

### Results of syntheses

The various outcomes worsened in four studies (33%), improved in one (8%), and there were no significant changes in two studies (16%) or unclear or unmeasured trends in five (42%). Particularly relevant to the purpose of understanding long-term changes, even with studies deemed to be eligible for this review, the duration or timing of follow-up was relatively short. In three articles (25%), health impacts lasting 1–12 months were considered, while three studies (25%) focused on health problems that lasted longer than one year. The duration of health issues for the remaining six studies (50%) is unclear and perhaps not explicitly intended to be a factor.

Target outcomes can be broadly categorized as either suicide or symptoms/diagnoses associated with various mental health outcomes (including hospitalization or admission to psychiatric facility). For the latter category, conditions included dementia praecox and PTSD as well as non-specific measures related to stress and quality of life. While the articles did not always explicitly attempt to statistically determine associations or causal relationships with potential causes, most proposed one or more mechanisms through which these outcomes might have occurred. Six (50%) of the articles suggest effects of the illness itself. We refer to this as a “direct” effect (Table I ). Other mechanisms (“indirect”) included social integration and distancing or disease containment strategies including isolation (three articles, 25%), working in health care (one article, 8%). Two articles (17%) suggested multiple possibilities or general experience and exposure to the pandemic.

As noted above, only the 1918 and 2009 pandemics were represented in these studies. Both suicide and symptoms/diagnoses associated with mental health outcomes were studied for each pandemic, although more of the 1918 studies focused on suicide than those for the 2009 pandemic (three of seven articles versus one of five). This difference is likely the result of differing data availability: high-quality, population-level reporting of less serious mental health conditions is rarely available from the early 20th century. As a result, studies have either focused on more serious outcomes, or have considered smaller localities or samples within broad populations when examining a wider range of outcomes.

We note that most studies focused on North American or European data, raising concerns about the impacts of geographic variation in pandemic experiences, as well as cultural variation in the understanding, diagnosis, and treatment of mental health conditions. These concerns do limit the external validity of our findings. As indicated in the protocol, we also intended to synthesize results based on whether studies revealed differences between those with and without pre-existing mental health conditions. However, most studies were conducted at the population level or did not report mental health status prior to the relevant pandemic, preventing adequate exploration of this question.

### Reporting biases

Our selection criteria limited the studies to English-language publications. Further potential reporting biases arise from methodological, disciplinary, and time elements. By focusing on published articles returned from database searches, other potential sources including books, white papers, or firsthand narratives, might not be adequately represented. Additionally, we excluded case studies because of concerns about anecdotal evidence from small sample sizes. However, as mental health outcomes and indeed pandemics themselves may be considered relatively rare phenomena, smaller sample sizes and case studies might be a more abundant source of evidence for this research question. Similarly, possibly owing to either the search or the screening strategies, most sources considered for this review might have been biased towards fields using quantitative or clinical methods rather than other approaches such as historical or qualitative analysis. Finally, as noted, the sources reported outcomes for the 1918 and 2009 influenza pandemics, a bias also seen in other pandemic-related systematic reviews [11].

### Certainty of evidence

All four studies that investigated suicide as an outcome found at least some support for increased rates during or following pandemics. The studies investigating symptoms, diagnoses and/or hospitalizations for various mental health outcomes were mixed and so are inconclusive.

## Conclusion

Based on a limited number of studies, this review suggests mental health consequences might have occurred following past influenza pandemics and persisted for some time. However, the review also highlights several gaps that should be addressed in future research. Notably, most studies included in this review lacked individual-level data and clear consideration of duration. Although studies covering short-term effects associated with acute infection were excluded, how long a condition must last to be considered as long-term is not clear. Similarly, the boundary between mental, emotional, and physical health is not distinct. Researchers should think more broadly about outcomes of interest. Clinical diagnoses of specific conditions are likely too restrictive—and might have changed over time—compared with the wide range of potential outcomes considered when developing the protocol for this review and identified by theoretical commentaries.

The extensive research into the mental health effects of COVID-19 and studies that combine the impacts of different types of infectious diseases show substantial evidence of mental health consequences, although the pathogen itself might not be the main mechanism as worsening psychiatric symptoms have been observed for both infected and non-infected individuals [12]. However, only the 1918 flu pandemic is a comparator of similar scale in terms of global morbidity and mortality, social disruption, and use of non-pharmaceutical interventions. Therefore, more research is needed to identify mental health considerations that are unique to a particular pandemic, especially for extremely disruptive pandemics, and to distinguish such outcomes from those that might recur across different contexts. Such studies should be particularly concerned with evidence of long-term mental health outcomes and their mechanisms. While the investigation of mechanisms was not the main goal of this study or of the articles reviewed, the causes of mental health morbidity from pandemics are worthy of much further investigation and comparative analysis between pandemics. New insights might be gained in the study of historical pandemics through effective identification and use of archival sources, such as those associated with institutions (e.g. admission records and annual reports of asylums, sanatoria, and schools) or administrative records (e.g. military populations or insurance companies).
